# Co-registered MR tissue phase mapping and speckle tracking echocardiography: inter-modality comparison of regional myocardial velocities in pediatric patients

**DOI:** 10.1186/1532-429X-17-S1-Q103

**Published:** 2015-02-03

**Authors:** Joseph Camarda, Patrick Magrath, Keyur Parekh, Varun Chowdhary, Cynthia K Rigsby, Michael Markl

**Affiliations:** 1 Ann & Robert H. Lurie Children's Hospital of Chicago, Chicago, IL, USA; 2 Northwestern University, Chicago, IL, USA

## Background

MR tissue phase mapping (TPM) enables assessment of regional myocardial velocities. The purpose of this study was to compare regional myocardial velocities obtained by both TPM and speckle tracking echocardiography (STE) to assess agreement and potential systematic differences between modalities.

## Methods

N=19 patients with congenital heart abnormalities and biventricular physiology who had undergone cardiac MRI including TPM and transthoracic echocardiography within 6 months of MRI were identified (mean age=14 (7-20) years). Average time between MR and echo studies was 2.3±1.4 months. MRI was performed on a 1.5T MR-system (Aera, Siemens, Erlangen, Germany). TPM were acquired data in short axis orientation using a black-blood prepared cine phase-contrast sequence with tri-directional phase encoding (venc=25cm/sec, temporal resolution=24msec, spatial res=2.9x2.4mm, slice thickness=8mm). Analysis included manual segmentation of the left ventricular contours and transformation of the acquired tri-directional velocities into radial, circumferential, and long-axis velocities.

Echocardiograms were reviewed retrospectively. Mid-chamber short axis images with adequate endocardial visualization were selected. 2-dimensional STE assessment was performed offline using TomTec Image Arena (version 4.3) with determination of peak radial velocities and time-to-peak (TTP) analysis for both systole and diastole. Assessments were performed during a single cardiac cycle with imaging frame rate of 30 frames per second.

To directly compare regional velocities, data from both modalities were mapped onto the mid-ventricular section of the AHA 16-segment model and systolic and diastolic radial peak velocities as well as TTP were calculated for each segment. Inter-modality differences for peak velocities and TTP were assessed using paired t-tests; p<0.05 was considered significant.

## Results

**Figure 1:**
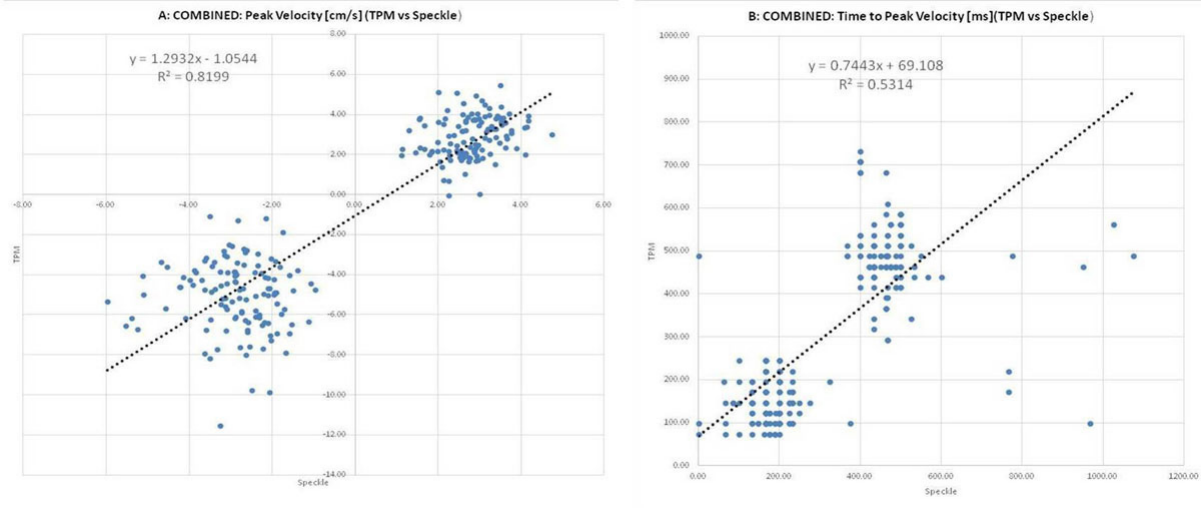
Correlation analysis of systolic and diastolic radial peak velocities (A) and systolic and diastolic TTP (B) for regional myocardial motion assessed by TPM and STE (TTP - Time to peak velocity; TPM - Tissue phase mapping; STE - Speckle tracking echocardiography).

Analysis of regional mid-ventricular systolic and diastolic velocity-time courses and extraction of peak and TTP velocities was successfully performed in all subjects. Comparison of regional velocities demonstrated good correlation (R^2^=0.82) (Figure 1a). Comparison of regional TTP showed a positive correlation, however, less strong (R^2^=0.53) (Figure 1b). Mean radial peak velocities (averaged over all segments and subjects) were similar for TPM compared to STE for systole (2.97±0.92 cm/s vs. 2.81±0.7 cm/s, p=0.097) but significantly higher by 81% for diastole (5.19±1.7 cm/s vs. 2.86±0.98 cm/s, p<0.001). TTP were higher for STE in systole, but similar in diastole (Table 1).

**Table 1:** Comparison of average peak and TTP velocities between TPM MRI and STE. (TTP - time to peak; TPM - tissue phase mapping; STE - speckle tracking echo; Std - standard deviation).

	Systole				Diastole			
	Peak Velocity [cm/s]		TTP [ms]		Peak Velocity [cm/s]		TTP [ms]	

	Mean	Std	Mean	Std	Mean	Std	Mean	Std

STE	2.81	0.70	192	120	-2.86	0.98	475	112

TPM	2.97	0.92	141	48	-5.19	1.70	476	62

p-value	0.097		<0.001		<0.001		0.918	

## Conclusions

Regional myocardial velocities in pediatric patients can be measured by TPM and results correlate well with STE. Comparisons of regional velocities are similar, but systematically underestimated by STE. Diastolic TTP showed no significant difference. Further studies are needed to identify factors that may contribute to these differences.

## Funding

None.

